# Assessment of oral health conditions presented in photographs - is there a difference between dentists and non-dental professional caregivers?

**DOI:** 10.1186/s12903-020-01171-x

**Published:** 2020-07-03

**Authors:** Stefanie Krausch-Hofmann, Trung Dung Tran, Dominique Declerck, Johanna de Almeida Mello, Anja Declercq, Emmanuel Lesaffre, Jan De Lepeleire, Joke Duyck

**Affiliations:** 1 KU Leuven Population Studies in Oral Health - Department of Oral Health Sciences, Kapucijnenvoer 7/a - box 7001, 3000 Leuven, Belgium; 2 KU Leuven Biostatistics and Statistical Bioinformatics Centre (L-BioStat) - Department of Public Health and Primary Care, Kapucijnenvoer 35/d - box 7001, 3000 Leuven, Belgium; 3 KU Leuven LUCAS - Centre for Care Research and Consultancy, Minderbroedersstraat 8 - box 5310, 3000 Leuven, Belgium; 4 KU Leuven CESO – Center for Sociological Research, Parkstraat 45 - box 3601, 3000 Leuven, Belgium; 5 KU Leuven Academic Centre for General Practice - Department of Public Health and Primary Care, Kapucijnenvoer 33/j - box 7001, 3000 Leuven, Belgium; 6 Biomaterials/BIOMAT - Department of Oral Health Sciences, Kapucijnenvoer 7/a - box 7001, 3000 Leuven, Belgium

**Keywords:** Oral health assessment, Oral photographs, Caregivers, Dentists

## Abstract

**Background:**

Photographs can help non-dental professional caregivers to identify problems when inspecting the mouth of care-dependent older individuals. This study evaluated whether the assessment of oral health-related conditions presented in photographs differed between dentists and non-dental professional caregivers.

**Materials and methods:**

One-hundred-and-seventy-nine photographs were taken from long-term care facility residents and from patients at the Department of Dentistry of a University Hospital. The following oral health aspects were depicted: denture hygiene, oral hygiene, teeth, gums, tongue and palate/lips/cheeks. Collection continued until for each oral health aspect a pool of photographs was available that showed conditions from perfect health and hygiene to severe problems. A segmented Visual Analogue Scale was applied to assess the conditions presented in the photographs. Each photograph was assessed by each participant of this study. The benchmark was established by three dentists with academic-clinical expertise in gerodontology, special needs dentistry and periodontology. For each photograph, they provided a collective score after reaching consensus. Photographs were assessed individually by 32 general dentists and by 164 non-dental professional caregivers. Linear mixed effects models and generalized linear mixed effects models were fitted and mean squared errors were computed to quantify differences between both groups.

**Results:**

For the different oral health aspects, absolute distances from the benchmark scores were 1.13 (95%CI:1.03–1.23) to 1.51 (95%CI:1.39–1.65) times higher for the caregivers than for the dentists. The odds to overestimate the condition were higher for the caregivers than the dentists for oral hygiene (OR = 0.72, 95%CI = 0.62–0.84) and teeth (OR = 0.74; 95%CI = 0.61–0.88). The odds to underestimate the condition were higher for the caregivers than the dentists for gums (OR = 1.39; 95%CI:1.22–1.59) and palate/lips/cheeks (OR = 1.22; 95%CI = 1.07–1.40). Over all assessments, the variance in caregiver scores was 1.9 (95%CI:1.62–2.23) times higher than that for the dentists.

**Conclusion:**

Small but significant differences were found between dentists and non-dental professional caregivers assessing oral health-related conditions presented in photographs. When photographs are used to aid non-dental professional caregivers with the oral health assessment, these visualizations should be complemented with comments to facilitate accurate interpretation.

## Background

International research shows that oral health in care-dependent individuals is poor [1–3]. This is confirmed by the ‘World Report on Ageing and Health’, which states that “Oral health is a crucial but often neglected area of healthy ageing” [4]. The challenges of oral disease are considerable due to associations with impaired oral functioning [5], aspects of general health [6–8], a number of systemic diseases [9–11] and quality of life [12–15]. Daily oral care and regular professional check-ups are the cornerstones of good oral health. They both are impeded in care-dependent individuals due to physical and cognitive restraints and insufficient availability or accessibility of care [5]. An oral health assessment by non-dental professional caregivers is suggested as a supplementary procedure to detect oral-health related care- and treatment needs [16]. A variety of assessment instruments for caregivers are available such as the Oral Health Assessment Tool (OHAT), the Revised Oral Assessment Guide (ROAG) or the oral health-related section of the Minimum Data Set/interRAI suite of instruments (MDS/interRAI). The instruments above expect caregivers to assess different oral health aspects on a nominal scale, in order to determine whether assistance with daily oral care and/or referral to an oral health professional is required. However, studies on concurrent validity that compare professional oral examination data with non-dental caregiver registrations show shortcomings in the latter in correctly identifying oral care needs [16–19].

As pictures do support, reinforce and illustrate written text [20], oral photographs could be used to visualize item categories and illustrate training materials. A review on the role of pictures in improving health communication concluded that visualizations can support comprehension by providing a context for organizing text information. Pictures are particularly helpful when content is complex and when prior knowledge of individuals is low [21]. Hence, clinical photographs may help non-dental professional caregivers to correctly identify oral care needs. However, expertise differences with regard to the interpretation of medical visualizations have been described in literature [22]. This raises the question of whether non-dental professional caregivers see what dentists see. Or, in other words, whether the interpretation of clinical photographs significantly differs between both groups.

To our knowledge the present study is the first to explore the presence of differences between dentists and non-dental professional caregivers assessing oral health-related conditions presented in photographs. A pool of clinical photographs showing different oral health aspects was used to test the following hypotheses:

1. Distance from a benchmark assessment is higher for professional non-dental caregivers than for dentists.
2. Direction of the distance from the benchmark depends on the oral health aspect that is shown.
3. Variance around the benchmark assessment is higher for professional non-dental caregivers than for dentists.

## Materials and methods

### Background of the research project

The present study is part of a larger research project that aims to develop an optimized photograph-supported oral health-related section for the interRAI suite of instruments (ohr-interRAI). The interRAI suite is used internationally and consists of tools for comprehensive assessment of conditions and needs of care-dependent individuals. Different versions are available for various sectors, such as home care, nursing homes, hospitals or mental health care settings [23].

### Collection of a pool of clinical photographs

Photographs were taken from consenting long-term care facility residents and from patients at the Department of Dentistry of the University Hospitals KU Leuven, Belgium. Equipment for professional digital dental photography was used: Canon EOS5500 camera, EF-S60mmF2.8USM Macro Objective Lens and a Macro Ring Lite MR-14EX. Lip and cheek retractors as well as oral mirrors were used.

High-definition close-up photographs were taken of the following oral health aspects: denture hygiene, oral hygiene, teeth, gums, tongue and palate/lips/cheeks. The dentist (SKH) who took the photographs used the criteria provided in Table 1 to ensure that for each oral health aspect a balanced number of photographs was included showing ‘acceptable’, ‘not acceptable moderate’ and ‘not acceptable marked’ conditions. In addition, each condition of the different oral health aspects included multiple variations such as full and partial dentures, anterior and posterior teeth as well as buccal and palatal/lingual views. Implant-supported structures were depicted and dorsal, lateral and ventral tongue photographs were taken.

**Table 1:** Verbal description of the segments of the Visual Analogue Scale

Oral health aspects	Segments of the VAS		
	1 Acceptable	2 Not acceptable, moderate	3 Not acceptable, marked
Denture hygiene: Part of the inner surface covered with dental plaque or tartar	< 1/3	1/3–2/3	> 2/3
Oral hygiene: Part of the surface of teeth or denture retainers covered with dental plaque or tartar	< 1/3	1/3–2/3	> 2/3
Teeth	All teeth sound, adequately filled, maybe tooth wear	≥ 1 tooth broken, with decay, defect fillings, root remnants	a
Gums	Pink and firm, maybe minor aberration in color or texture	Moderate redness, swelling, glassy	Marked redness, swelling, bleeding, sores, wounds, fistulas
Tongue	Small bumps on upper and lateral surface, moist, pink	General redness, patches, extensive coating, deep grooves, dry	Red and/or white lesions, swelling, sores, wounds
Palate, oral surface of lips and cheeks	Smooth, moist, pink	General redness, rough, dry	Red and/or white lesions, swelling, sores, wounds

The criteria provided in this table were developed based on a review of the literature and several discussion rounds among the members of the research group

^a^For teeth, definition of the appearance of ‘not acceptable marked’ conditions was not considered meaningful

*Verbal description of the appearance of dentures and oral tissues for each segment of the Visual Analogue Scale that was applied for assessment of the photographs*

The final pool consisted of 179 clinical photographs: denture hygiene (30), oral hygiene (30), teeth (20), gums (35), tongue (30) and palate/lips/cheeks (34) including a variety of conditions from perfect health and hygiene to severe problems.

### Assessment of the photographs

**Fig. 1:**
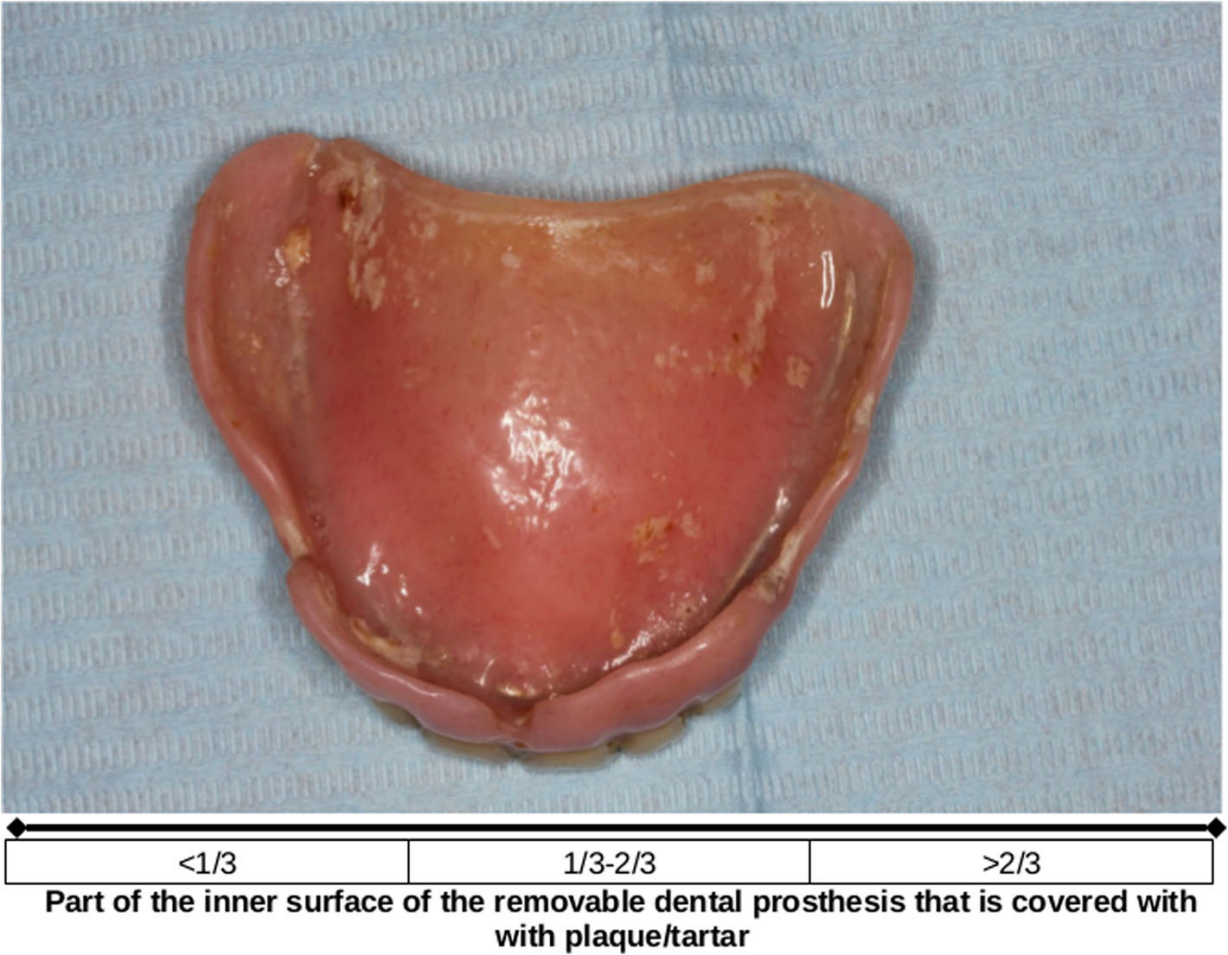
Example of a photograph on denture hygiene and the scale that was applied

Each of the 179 photographs was assessed by each participant of this study. Photographs were presented per oral-health aspect in six blocks in randomized order on an individual PC screen that was placed in front of each participant. A three-segmented 150 mm Visual Analogue Scale (VAS), (100 mm two-segmented for teeth) was used for the assessment. Participants were instructed to apply the VAS as follows: 1. First select a segment of the scale. The definition of the segment should most suitably describe the appearance of denture or tissue shown on the photograph (Table 1). 2. Then indicate a position on the VAS that is located within the range of the chosen segment. Zero on the VAS represents a perfectly healthy or clean condition, while the right end of the scale indicates severe problems. An example of a photograph on denture hygiene and the scale that was applied is shown in Fig. 1.

The benchmark was established by three dentists with expertise in gerodontology, special needs dentistry and periodontology, affiliated with the Dentistry Department of the University Hospitals, KU Leuven. For each photograph they provided a collective VAS score after reaching consensus. The photographs were assessed individually by dentists and by non-dental professional caregivers in separate sessions. The sessions were organized during certified continuous education activities that were in no way linked to the assessment of the photographs. Participants did not receive any training related to appearance, diagnosis or interpretation of oral health-related pathology.

### Sample and recruitment of dentists and non-dental professional caregivers

Previous studies with a similar aim and design were not available to estimate the minimum sample size. The current research focused on large effects as literature reports substantial problems of professional non-dental caregivers to correctly identify oral treatment need in care clients [18, 19, 24]. Defining a standard α-level of 0.05 and a recommended power of 0.8 to compare mean differences, a minimum of 26 participants per group were required to detect a large effect (0.8) [25].

To recruit dentists, an invitation was sent to all attendees of previous permanent education activities organized by the Department of Oral Health Sciences of the University. To recruit the caregivers, care facilities, high-schools for nursing education, umbrella organizations and professional associations for caregivers in Flanders, Belgium were contacted to circulate the invitation among employees or members. Professional non-dental caregivers having direct contact with clients in home- or long-term care were invited to participate (e.g., nurses, auxiliary nurses, speech therapists, occupational therapists, dietitians or physicians). Participation was allowed to all dentists and non-dental professional caregivers who responded to the invitation.

### Statistical analysis

The absolute value of the distance from the benchmark score was calculated for dentists and for non-dental caregivers for each photograph. Due to skewness of the distribution, logarithm of the values was used. The direction of distance from the benchmark indicated whether the score assigned to a photograph was lower, equal or higher than the benchmark score.

To quantify differences between caregivers and dentists with regard to the distance from the benchmark as well as the direction of this difference, a linear mixed effects model and a generalized linear mixed effects model were fitted, respectively. Type of assessor (caregiver, dentist) and oral health aspect (denture hygiene, oral hygiene, teeth, gums, tongue, palate/lips/cheeks) were added to the models as random effects.

Mean squared errors were computed to compare the scores provided by non-dental caregivers and dentists with regard to the variance around the benchmark for each oral health aspect. Statistical programs R (version 3.6) and SAS (version 9.4) were used.

## Results

### Characteristics of dentists and caregivers

Thirty-two dentists and 164 non-dental professional caregivers participated in this study. All participants were Caucasian. Table 2 shows that most participants were female. In the caregiver group the gender imbalance was more pronounced with 94.5% female participants. The caregiver group was also relatively younger than the dentist group. Most frequent occupations among caregivers were nurses and nurse aids with 57.9 and 23.8%, respectively. All dentists were primary dental care providers with 46.9% having an additional training in endodontology or prosthetic dentistry.

**Table 2:** Characteristics of dentists and non-dental caregivers

		Dentists, *N* = 32	Caregivers, *N* = 164
Gender in %	Female	68.8	94.5
	Male	31.2	5.5
Age groups in %	< 30 years	9.4	29.3
	30–40 years	12.5	22.6
	41–50 years	31.3	37.8
	> 50 years	46.9	10.4
Occupation	Nurse		57.9
	Nurse aid		23.8
	Nurse lecturer		7.3
	Speech therapist		3.7
	Others (e.g., physician, psychologist, dietitian)		7.3

### Distance from the benchmark

**Fig. 2:**
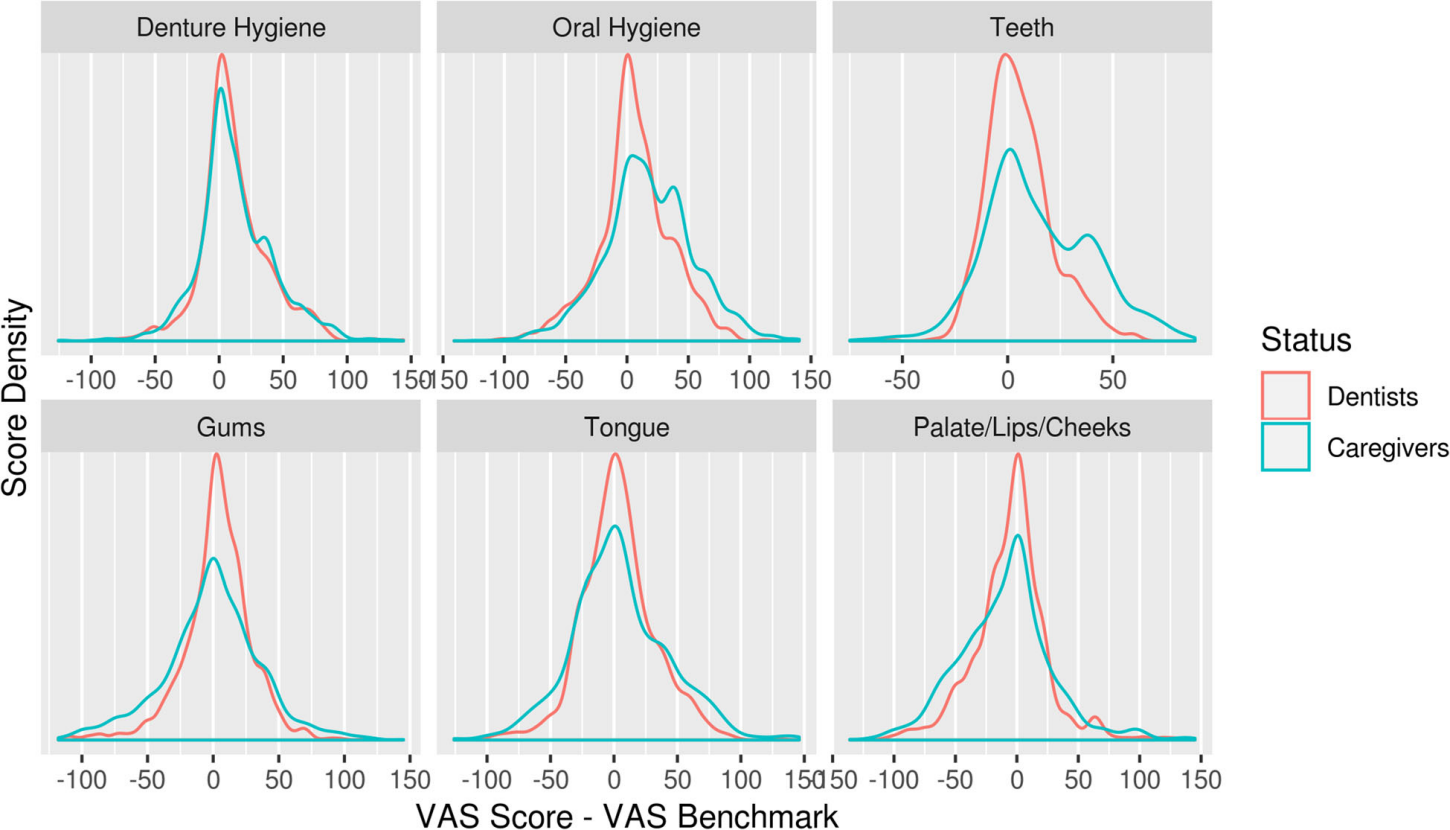
Density plots to illustrate distances from the benchmark scores for dentists and caregivers

Figure 2 illustrates the distance from the benchmark for dentists and caregivers, for each oral health aspect, respectively. In both groups, the majority of the assessments peak around the value zero, indicating no or little distance from the benchmark. However, in each of the six graphs, the curve of the dentists exceeds the curve of the caregivers around the zero value. This implies that the distance from the benchmark tends to be lower for dentists. Accordingly, over all photographs the mean absolute distance from the benchmark score on the VAS is 20.1 for the dentists and 27.7 for the caregivers

Table 3 presents the results of the linear mixed effects model. It confirms that for all oral health aspects the distances from the benchmark scores are 1.13–1.51 times higher for the caregivers than for the dentists.

**Table 3:** Distance from the benchmark

Oral health aspect	Distance from the benchmark: difference between dentists and caregivers on log scale (increase for caregivers)	95% confidence interval	*P*-value
Denture hygiene	0.118 (1.13)	0.028–0.208	0.010
Oral hygiene	0.326 (1.39)	0.236–0.416	<.0001
Teeth	0.411 (1.51)	0.308–0.513	<.0001
Gums	0.414 (1.51)	0.328–0.499	<.0001
Tongue	0.368 (1.44)	0.278–0.458	<.0001
Palate, lips, cheeks	0.347 (1.41)	0.260–0.435	<.0001

*For all oral health aspects the distances from the benchmark scores were significantly higher for the caregivers than for the dentists*

### Direction of the distance from the benchmark

With regard to the direction of the distance, a lower VAS score than the benchmark implies that the condition was underestimated by the participant. A higher score than the benchmark implies that the condition was overestimated. Table 4 provides an overview over all photographs for caregivers and dentists.

**Table 4:** Direction of the distance from the benchmark – overview over all photographs

Direction of the distance from the benchmark	Dentists in %	Caregivers in %
Lower score than benchmark (= underestimation)	40.62	41.87
Same score as benchmark	3.37	0.78
Higher score than benchmark (= overestimation)	56.02	57.35

The results of the generalized linear mixed effects model allow a more detailed view. Table 5 illustrates the odds ratios for caregivers versus dentists to assign a lower score than the benchmark. Odds ratios are < 1 for aspects of hygiene and teeth, but > 1 for aspects of the oral soft tissues. This implies that compared to the dentists, caregivers tended to overestimate aspects of hygiene and condition of the teeth, but underestimated aspects of the soft tissues.

**Table 5:** Direction of the distance from the benchmark

	Underestimation of the condition (lower score than benchmark, odds ratio caregivers/dentists)	95% confidence interval	*P*-value
Denture hygiene	0.95	0.81–1.11	0.522
Oral hygiene	0.72	0.62–0.84	<.0001
Teeth	0.74	0.61–0.88	0.001
Gums	1.39	1.22–1.59	<.0001
Tongue	1.12	0.97–1.30	0.118
Palate/lips/cheeks	1.22	1.07–1.40	0.004

*For oral hygiene and teeth the odds to assign a lower score than the benchmark were significantly higher for the dentists. For gums and palate/lips/cheeks the odds to assign a lower score than the benchmark were significantly higher for the caregivers*

### Variance around the benchmark

Considering all oral health aspects, the variance around the benchmark scores was significantly higher for the caregivers than for the dentists. The mean squared error was 1.9 times higher for the caregivers than for the dentists (95% confidence interval: 1.62–2.23). When mean squared errors were computed for each oral health aspect separately, no differences were found between the two groups.

## Discussion

### Interpretation and relevance of the study results

To our knowledge this is the first study that evaluated whether the assessment of oral health-related conditions presented in clinical photographs differed between dentists and non-dental professional caregivers. Results indicate small but significant differences. A first graphical analysis illustrated that in both groups the majority of the assessments peaked around the benchmark scores. Graphs of dentists and caregivers appeared approximately congruent. This is in line with two studies from the field of dermatology showing high accuracy of nurses to classify skin damage shown in photographs when compared to dermatologists [26, 27].

A more detailed evaluation of the data revealed small but significant differences between both groups, with caregivers having a higher distance from the benchmark than dentists. In addition, the variance in scores provided by the caregivers was higher than the variance in scores provided by the dentists. This confirms the findings published by Yazdanyar et al. (2013) who compared general practitioners and dermatologists with regard to their congruence with a benchmark in identifying acne morphology using photographs and a short description. In all cases, responses of the dermatologists were more congruent with the benchmark and variation was lower compared to the general practitioners [28]. A meta-analysis on differences in the comprehension of visualizations found higher performance accuracy and shorter reaction times for experts than for non-experts [29].

With regard to the direction of the distance from the benchmark, differences between dentists and caregivers depended on the type of oral health aspect. For photographs showing aspects of oral hygiene and teeth, the non-dental professional caregivers tended to overestimate the condition compared to the dentists. In contrast, for photographs showing gums and palate/lips/cheeks the odds to underestimate the condition were higher for the caregivers than for the dentists. To suggest a possible explanation, age-related physiological changes such as discoloration or tooth wear might be misinterpreted by non-dental professional caregivers. Furthermore, dental plaque and tooth decay may look impressive to non-dental caregivers, while dentists know that these conditions often can be treated easily. On the other hand, caregivers are maybe less aware than dentists that oral soft tissue lesions can involve harmful malignities.

Considering the supporting role of pictures in health communication, our findings confirm that complex visualizations require instruction and guidance to ensure a correct interpretation [21]. Hence, when photographs are used to aid non-dental caregivers with the oral health assessment, these visualizations should be complemented with comments to facilitate accurate interpretation. Results of the current study indicate that for aspects of hygiene and teeth, instructions are needed to differentiate between pathology and normal, age-related phenomena. It should further be emphasized that soft tissue lesions require close attention.

With regard to the development of the optimized interRAI oral health-related section, the benefits of including visualizations might reach beyond the correct identification of care needs. Focus group discussions with caregivers revealed that oral health has low priority in the care environment and that the oral health-related section of the interRAI is completed only superficially without inspection of the mouth [30]. As human beings are attracted to visual stimuli [20], photographs have the potential to enhance attention and raise awareness for the oral health assessment. Inclusion of various views of the different oral health aspects - such as dorsal, lateral and ventral tongue photographs - might motivate a more thorough assessment of the mouth. In this context it needs to be mentioned that oral health is often neglected in current training programs for non-dental professional caregivers. A study showed that among high schools in Norway providing basic education for auxiliary nurses, solely 49% offered three or more hours of teaching on oral health [31]. In a French study with professional caregivers only 21% reported previous theoretical training on oral disorders [32]. More emphasis on the topic of oral health during professional training of non-dental caregivers may raise the awareness and improve the ability to correctly recognize pathology.

### Study limitations and further research

Correct understanding of the terminology used to describe the different segments the VAS can be questioned for certain participants such as care aides who often receive only limited professional training. Accordingly, involvement of non-dental professional caregivers in development and pilot-testing of the scale should have been considered. Although the minimum sample size was exceeded for both groups, the number of participants was markedly unbalanced. The sample reflected actual differences between dentists and non-dental professional caregivers with regard to gender and age, but was not representative for the population of both groups. Further research – preferably on an international scale - needs to clarify whether findings are generalizable. In addition, the impact of participant demographics and oral health-related training, knowledge and awareness should be evaluated.

## Conclusion

Small but significant differences were found between dentists and non-dental caregivers regarding the assessment of oral health-related conditions presented in clinical photographs. When photographs are used to aid non-dental professional caregivers with the oral health assessment, these visualizations should be complemented with comments to facilitate accurate interpretation.

## Data Availability

All data analyzed during this study are included in this published article [and its supplementary information files].
